# Advances in Translational Regenerative Therapies

**DOI:** 10.3390/jcm12082838

**Published:** 2023-04-13

**Authors:** Harriet Kiwanuka, Alice T. Wang, Dennis P. Orgill

**Affiliations:** 1 Division of Plastic and Reconstructive Surgery, Brigham and Women’s Hospital, Boston, MA 02115, USA; 2 Harvard Medical School, Boston, MA 02115, USA

## 1. Introduction

Regenerative medicine aims to replace damaged cells and tissues following injury. Many recent advances in regeneration research have occurred within the integument. Advancements in this field require innovation and translation into clinical practice. This process is complicated by variability in human conditions and the realities of the clinical environment, with specific barriers including the potential for infection, mechanical disruptions, and human immune responses to regenerative therapies.

We review five recent papers in the *Journal of Clinical Medicine* related to advances in regenerative medicine with a range of clinical applications. We discuss exciting new advances in this field, with foci on cell-based therapies, biomolecular treatments, and tissue engineering applications. There is a need for not only robust proof of concept studies, but also clinical studies that demonstrate both the safety and efficacy of these products to receive regulatory approval. Each of these products needs to exist within the economic environment of our healthcare system as defined by our political structures.

The phases of wound healing are hemostasis, inflammation, proliferation, and remodeling [1]. These phases are frequently studied in the skin, but analogous processes occur in other tissues. Each phase is characterized by a predominant cell type. Platelets initiate the clotting process during hemostasis. Neutrophils initiate the inflammatory phase, then macrophages critically influence wound healing during this phase [2]. Fibroblasts consequently synthesize collagen rapidly during the proliferative stage. Within remodeling, collagen creation continues as keratinocytes begin re-epithelization [3].

## 2. Cell-Based Therapies

Cell-based therapies enable key biological processes for enhanced wound healing. Domaszewska-Szostek et al. [4] set forward a review of different cell-based therapies for burn care. Their analysis details findings from 49 studies, with an emphasis on keratinocyte and fibroblast-based therapies (38 of the 49 studies) for the treatment of partial and full thickness burns. These studies included not only experimental autologous engineered skin, but also commercially available products such as autologous keratinocyte wound dressings (Epicel^®^ Vericel Corporation, Cambridge, MA, USA) and keratinocyte with fibroblast cellular spray (Keraheal™ Biosolution Co. Ltd, Seoul, Korea, ReCell^®^ Avitas Medical Associates, Valencia, CA, USA). Many of the studies reported promising results, showing enhanced wound healing, improved scar character, decreased length in hospital stay, and improved survival both with fibroblast or keratinocyte therapies alone and in combination with skin grafting. However, most of the studies (31/49) had no robust control to assess whether the novel therapy surpassed the standard of care. Many of the studies’ endpoints were solely to demonstrate efficacy, with outcomes such as graft “take rate”, time to epithelization, and quality of regenerated epidermis. The lack of uniform metrics to evaluate important variables such as epithelization, graft take, and hypertrophic scarring precludes comparison among studies and represented therapies. Additionally, although there are promising findings in the utilization of cell-based therapies, the predominant barriers to use them include cost, shelf life, insufficient donor sites, and difficulty of use. Hopefully, future randomized comparative trials can be performed to better inform clinicians of the best treatment strategy for each clinical problem.

## 3. Biomolecular Therapies

Adjunctive therapies can also play a pivotal role in wound healing. Russo et al. [5] performed a single-center, parallel, randomized trial with 60 patients to evaluate the efficacy and tolerability of the products Connettivina^®^ Bio Plus (Fidia Farmaceutici S.p.A., Abano Terme, Italy) and Fitostimoline^®^ Plus (Farmaceutici Damor S.p.A., Napoli, Italy). Connettivina^®^ is a hyaluronic acid and silver sulfadiazine cream. Hyaluronic acid is a glycosaminoglycan (GAG) that is involved in all phases of wound healing. It stimulates inflammatory cells migration, differentiation, and proliferation [6]. It also regulates extracellular matrix organization and metabolism. Fitostimoline Plus is made from an antioxidant polysaccharide extracted from wheat plant Triticum vulgare (Rigenase) as well as polyhexanide. Rigenase^®^ (Farmaceutici Damor S.p.A., Napoli, Italy) has been found to stimulate extracellular matrix creation and enhance keratinocyte activity. The primary endpoint of the study was the rate of wound healing, defined as the rate of reduction in the wound area from the initial baseline visit. They found that Fitostimoline^®^ Plus (Farmaceutici Damor S.p.A., Napoli, Italy) was more effective at treating acute skin wounds than Connettivina^®^ Bio Plus, (Farmaceutici Damor S.p.A., Napoli, Italy) as demonstrated by an increased reduction in wound area, wound healing rate, and fibrin in wound bed. A limitation of this study is the classification of surgical wounds, burn wounds, and traumatic wounds under the umbrella term “acute skin wounds”. The mechanism of injury of wounds plays a huge impact on wound healing. For example, burn wounds have a more robust, prolonged inflammatory phase in comparison to incisional wounds [7]. Categorizing dissimilar wounds together and having a different distribution of wound types in each treatment group potentially influences the perceived wound healing rate. In future studies, standardization of wound mechanism of injury is crucial to have reproducible, universally applicable results.

## 4. Tissue Engineering

Therapies that employ the fundamentals of tissue engineering utilize stem cells, extracellular scaffolds, and stimulatory factors to promote wound healing. Stem cells are intrinsically pluripotent and can differentiate into native tissue based off their environment. Scaffolds replicate the extracellular matrix, allowing cells to repopulate and replace absent tissue. Stimulatory factors are growth factors that mimic physiologic processes when the body deploys cytokines and factors to instruct cells where, when, and how to grow. Asahina et al. [8] applied these principles and looked at the safety and efficacy of bone tissue engineering for patients with atrophic alveolar bone. They used bone marrow stromal cells as the stem cell, tricalcium phosphate granules as the scaffold, and platelet-rich plasma as the stimulatory factor. They found that within eight-years of follow-up, it was feasible and safe to use their model of tissue engineering for treating atrophic alveolar bones. Bone regeneration was observed in all eight patients, with 27 out of 29 dental implants integrated. A major limitation of the study was a lack of control groups to discern whether the scaffold, stem cell, or factor alone could have caused the observed outcome. Another limitation is the study size, as two patients were excluded due to cell protocol violations and bacterial contaminations. In future studies, proper control groups and a larger number of participants are required to characterize both the efficacy and mechanism of action of the product more confidently.

Barriers in tissue engineering include the effective delivery of stimulatory factors, the creation of effective scaffolds that are readily integrated biologically, and access to stem cells. Addressing the barrier to scaffold creation, Saini et al. [9] offered a review of 3D printing technologies that can be applied to create scaffolds using biomaterials and living cells in cardiovascular, integumentary, and musculoskeletal contexts. Using bio-inks, biomaterials such as natural polymers (collagen, fibrin, proteoglycans), and native cells creates a higher likelihood that tissue will be incorporated given its immunotolerance and mimicry of endogenous tissue. Challenges associated with bioprinting include, but are not limited to, a lack of stability of bio-fabricated tissue in vivo, the cost and expertise associated with bio-ink creation and 3D printing, and the small sizes of bio-printed tissues.

Savkovic et al. [10] addressed stem cell access by proposing the usage of mesenchymal stem cells from the hair follicle outer root sheath (MSCORS) compared to adipose mesenchymal stem cells. MSCORS were obtained non-invasively through plucking hair; adipose stem cells were obtained from discarded tissue from surgical procedures. In comparison to adipose mesenchymal stem cells, MSCORS have a higher capacity to promote angiogenesis by differentiating into endothelial and smooth muscle cells as demonstrated by their in vitro analysis. Additionally, MSCORS have limited donor site morbidity compared to adipose stem cells. Despite promising preliminary results, future in vivo studies are required to fully characterize the ultimate effects of MSCORS, including their dissemination throughout living tissue, differentiation into more intricate vascular structures, and longevity in the tissue.

Lastly, there are also studies investigating bioengineered artificial skin substitutes, aiming to improve the clinical utility of engineered tissue. Quinones-Vico et al. [11] examined the impact of antiseptics on the cell viability, structural integrity, and epidermal barrier function of bioengineered artificial skin substitutes consisting of keratinocytes and dermal fibroblasts. They applied the following antiseptics every 48 h for a 28-day period: Colistin, chlorhexidine digluconate, sodium chloride, and polyhexanide. They found chlorhexidine digluconate and polyhexanide significantly degraded bioengineered epithelium. Additionally, cell viability was most impacted by chlorhexidine.

## 5. Summary

In summary, the field of regenerative medicine is an evolving field that requires innovation and investigation for continued advancement. Principles of wound healing and tissue engineering will be at the crux of all future research for sustained discovery. Limitations of cell-, biomolecule-, and tissue engineering-based therapies persist. These limitations include the cost and safety of storage, the morbidity associated with harvesting autogenic cells, as well as the effective delivery and creation of stimulatory factors and scaffolds. Robust studies that rigorously evaluate the therapeutic potential of these treatments are required to improved efficacy compared to conventional therapies.

Furthermore, carefully designed studies are required for novel autologous and allogeneic products to obtain regulatory approval for global use. Within the past two decades, there has been an international increase of regenerative medicine-based therapies, driving regulatory bodies to determine the safest pathway for commercialization of these products. Starting in 2007, the European Union’s European Medicines Agency, the United States (Food and Drug Administration), Canada (Health Canada), Australia, (The Therapeutic Goods Administration), Japan (Ministry of Health, Labor and Wealth) and South Korea (Ministry of Food and Drug Safety) have constructed distinct pathways and legalities for the regulation of regenerative medicine products [12].

General regulatory pathways are similar among different countries’ regulatory agencies. All products are evaluated in three main stages: discovery, clinical research, and pre-approval request. The discovery stage consists of the basic scientific and preclinical animal studies. The clinical research stage encompasses clinical trials. For autologous and allogeneic products, clinical trials are commonly designed as randomized, controlled, comparative trials, combined phase I (safety) and phase II (effectiveness) trials, followed by carry phase III trials (comparison to existing treatment). All data are then submitted in a standardized format to the regulatory agency during the pre-approval request stage. The agency then decides whether the product is eligible for marketing authorization and/or a product license.

Although regulatory pathways are similar internationally, the classification and thus regulation of the common products greatly differ. With continued innovation in cell and tissue harvesting, expansion, and manipulation, there will be increased ambiguity regarding what products require which regulation under present-day laws (Hourd 2014) [13]. As the field evolves with cutting-edge advancements on the horizon, it remains imperative that we develop universal regulatory guidelines for the uniform evaluation of novel regenerative therapies.
